# Observational Study of Peritoneal Washing Cytology-Positive Gastric Cancer without Gross Peritoneal Metastasis in Patients who Underwent Radical D2 Gastrectomy

**DOI:** 10.1038/s41598-020-66637-y

**Published:** 2020-06-12

**Authors:** Hyun-Jeong Shim, Hyeon-Jong Kim, Seung Hyuk Lee, Woo-Kyun Bae, Eu-Chang Hwang, Sang-Hee Cho, Ik-Joo Chung, Hyun-Jin Bang, Jun Eul Hwang

**Affiliations:** 1 Department of Hematology-Oncology, Gwangju, Korea; 2 Department of Urology, Gwangju, Korea; 3 Chonnam National University Medical School and Hwasun Hospital, Gwangju, Korea

**Keywords:** Gastrointestinal cancer, Gastric cancer

## Abstract

Background The clinical features and therapeutic strategies for gastric cancer with positive peritoneal washing cytology but without visible gross peritoneal metastasis have not been defined. The aim of this study was to evaluate the effect and clinical prognostic value of postoperative chemotherapy in gastric cancer patients with positive peritoneal washing cytology without gross peritoneal metastasis who underwent radical D2 gastrectomy in terms of disease-free survival (DFS) and overall survival (OS). Materials and Methods Intraoperative peritoneal washing cytology was performed in 285 patients who underwent radical D2 gastrectomy between April 2004 and May 2016. Of them, 88 patients with positive cytology but without gross peritoneal metastasis were included in the study. In total, 64 patients received postoperative chemotherapy, whereas 24 patients underwent surgery only. Results Most gastric cancer patients with positive cytology without gross peritoneal metastasis demonstrated pT4 and/or pN3 disease. Postoperative chemotherapy improved DFS and OS compared to surgery only in gastric cancer patients with positive cytology without gross peritoneal metastasis (median DFS 11.63 vs. 6.98 months, *p* < 0.001; median OS 25.50 vs. 12.11 months, *p* < 0.001). In multivariate analyses of gastric cancer patients with positive cytology without gross peritoneal metastasis, no chemotherapy was the strongest clinical factor for poorer DFS (hazard ratio [HR] 3.76, *p* < 0.001) or OS (HR 4.37, *p* < 0.001). Conclusion Postoperative chemotherapy improves the survival outcome compared to surgery alone in gastric cancer patients with positive peritoneal washing cytology but without visible gross peritoneal metastasis who underwent radical D2 gastrectomy.

## Introduction

Although the incidence of gastric cancer has been decreasing in developed countries, it remains the fifth most common cancer and the third leading cause of cancer mortality worldwide^1^. In Korea, gastric cancer ranks second in cancer incidence and third in cancer mortality^2^. The treatment of choice for locally advanced gastric cancer in Asian countries, including Korea and Japan, is radical surgery followed by adjuvant chemotherapy^3–6^.

Peritoneal metastasis is the most frequent site of gastric cancer recurrence or metastasis and is associated with a very dismal prognosis^7,8^. The treatment options for advanced gastric cancer with overt gross peritoneal metastasis are only palliative systemic chemotherapy with or without surgical resection and/or intraperitoneal chemotherapy^9^.

Staging laparoscopy and peritoneal washing cytology have been evaluated for patients with gastric cancer to identify occult metastatic disease that is not detected by preoperative cross-sectional imaging, and positive peritoneal washing cytology in the absence of visible gross peritoneal implants is considered to be a poor prognostic factor for advanced disease and early recurrence and is defined as pM1 disease^10,11^.

The clinical features and therapeutic strategies for gastric cancer with positive cytology but without visible gross peritoneal metastasis have not been fully defined. The present study evaluated the effect and prognostic value of postoperative chemotherapy in patients with positive cytology but without gross peritoneal metastasis who underwent radical D2 gastrectomy in terms of disease-free survival (DFS) and overall survival (OS).

## Methods

### Patients

Intraoperative peritoneal washing cytology was performed in 285 patients who underwent radical D2 gastrectomy between April 2004 and May 2016. Of them, 88 patients with positive cytology but without gross peritoneal metastasis were included in the study. In total, 64 patients received postoperative chemotherapy and 24 patients underwent surgery only. Data from these patients were collected from our institutional database, and the survival data were updated at the time of analysis. The inclusion criteria were: patients with gastric adenocarcinoma who underwent radical gastrectomy and D2 lymph dissection with positive peritoneal washing cytology but without visible gross peritoneal metastasis. Patients with metastatic disease and patients with microscopically resection margin tumor-positive or macroscopically tumor-positive disease were excluded^12^. The Institutional Review Board of Chonnam National University Hwasun Hospital approved this study. All the procedures performed in this study involving human participants were in accordance with the ethical standards of the institutional review board at Chonnam National University Hwasun Hospital in Jeonnam, Korea, and with the 1964 Helsinki Declaration and its later amendments or comparable ethical standards. Informed consent was obtained from all individual participants included in the study^12^.

### Postoperative chemotherapy

We recommended postoperative chemotherapy for 85 patients excluding 3 patients; 2 patients are very old (>85) and 1 patient had several co-morbidities (chronic kidney disease and heart failure). 21 patients refused the chemotherapy. We administered postoperative chemotherapy with TS-1 (Taiho Pharmaceutical, Tokyo, Japan), oxaliplatin plus capecitabine (Xelox), oxaliplatin plus 5-fluorouracil (5-FU) (FOLFOX), or cisplatin plus TS-1 (CS) according to the physician’s judgement and patient preference. The Xelox regimen was administered every 3 weeks, and consisted of capecitabine (1,000 mg/m^2^ twice daily on days 1–14) plus intravenous oxaliplatin (130 mg/m^2^ on day 1)^13^. The TS-1 dose was determined based on body surface area (BSA). Patients received one of the following doses, divided in two, after meals daily: 80 mg for patients with a BSA < 1.25 m^2^, 100 mg for those with a BSA of 1.25–1.49 m^2^, and 120 mg for those with a BSA ≥ 1.50 m^2^. TS-1 was administered for 4 weeks followed by a 2-week rest period. TS-1 was administered for 1 year after surgery or until recurrence according to the physician’s judgement and patient preference^12,14^. The FOLFOX regimen was administered every 2 weeks, and consisted of intravenous oxaliplatin (85 mg/m^2^ on day 1), and leucovorin (200 mg/m^2^ on day 1), followed by 5-FU (2,600 mg/m^2^ intravenous continuous infusion over 24 h on day 1)^15^. TS-1 was given orally twice daily for the first 2 weeks of a 3-week cycle for patients on the CS regimen. The TS-1 dose was determined based on BSA, as described above. Cisplatin was given as an intravenous infusion of 60 mg/m^2^ on day 1^16^. Postoperative chemotherapy was administered for 6 months; however, in cases of Xelox, FOLFOX, and CS, capectabine, 5-FU, and TS-1 were administered over 6 months and/or until recurrence^12^.

### Follow-up

A physical examination, chest radiography, complete blood count, and biochemical tests were performed before each chemotherapy cycle. Computed tomography scans were performed every 2 months during the chemotherapy period and every 4 months thereafter until 5 years after surgery to assess tumor recurrence. If clinical signs or symptoms suggested clinical recurrence or the development of a new gastric cancer, further investigation was performed to determine whether the patient was disease free^12^.

### Statistical analyses

OS was defined as the time from the date of surgery to the date of death. DFS was defined as the time from the date of surgery to the date of recurrence or death, whichever occurred first. If neither event had occurred at the time of analysis, the patient was censored. Survival curves were estimated using the Kaplan-Meier method, and survival times were compared using the log-rank test. Factors associated with OS and DFS were identified by univariate and multivariate Cox proportional hazard regression models with hazard ratios (HRs) and 95% confidence intervals (CIs). Differences were detected using the chi-square test or Fisher’s exact test for categorical data and the *t*-test or the Mann-Whitney U test for continuous data. Statistical analyses were performed using SPSS version 24.0 (IBM Corp., Armonk, NY, USA) and R (R Foundation for Statistical Computing, Vienna, Austria; http://www.R-project.org) software. All P-values were two-sided, and P < 0.05 was considered significant^12^.

## Results

### Patient characteristics

The clinicopathological characteristics of the gastric cancer patients with positive cytology but without visible gross peritoneal metastasis (n = 88) are shown in Table 1. All of the patients were M1 disease (positive cytology). A total of 64 patients in chemotherapy group were comprised of 8 (12.5%) patients with T1/2/3 tumor, 56 (81.5%) with T4, 13 (20.3%) with N0/1/2 status, and 51 (79.7%) with N3. A total of 24 patients in surgery alone group were comprised of 1 (4.2%) patients with T1/2/3 tumor, 23 (95.8%) with T4, 3 (12.5%) with N0/1/2 status, and 21 (87.5%) with N3. The administered chemotherapy regimens were FOLFOX (n = 24), Xelox (n = 22), CS (n = 13), and TS-1 (n = 5). No significant differences were observed between the surgery, the postoperative chemotherapy group, or the surgery alone group in terms of age, sex, tumor location, Lauren classification, T stage, N stage, or perineural invasion. Most patients demonstrated T4 (chemotherapy vs. surgery alone, 81.5% vs. 95.1%) and N3 (chemotherapy vs. surgery alone, 79.7% vs. 87.5%) in both treatment groups. The chemotherapy group included more patients with a poorly differentiated/undifferentiated tumor grade and positive lymphovascular invasion (LVI+).

**Table 1:** Baseline characteristics of gastric cancer patients with positive cytology but without gross peritoneal metastasis treated with surgery and chemotherapy and those treated with surgery alone.

Variables, n (%)	Cytology (+)		P-value
	Chemotherapy (+)	Surgery alone	
	n = 64 (%)	n = 24 (%)	
Age (years)			
<61	35 (54.7)	8 (33.3)	0.076
≥61	29 (45.3)	16 (66.7)	
Sex			
Male	47 (73.4)	19 (79.2)	0.582
Female	17 (26.6)	5 (20.8)	
Tumor location			
GEJ, whole stomach	25 (39.1)	8 (33.3)	0.623
Body, antrum	39 (60.9)	16 (66.7)	
Tumor grade			
Well/moderately differentiated	11 (17.2)	10 (41.7)	0.017
Poorly/un-differentiated	53 (82.3)	14 (58.3)	
Lauren classification			
Intestinal	20 (31.3)	11 (45.8)	0.205
Non-intestinal (diffuse or mixed)	44 (68.8)	13 (54.2)	
T stage			
T1/2/3	8 (12.5)	1 (4.2)	0.253
T4	56 (81.5)	23 (95.8)	
N stage			
N0/1/2	13 (20.3)	3 (12.5)	0.401
N3	51 (79.7)	21 (87.5)	
LVI + /LVI−	53 (82.8)/11 (17.2)	13 (54.2)/11 (45.8)	0.006
PNI + /PNI−	57 (89.1)/7 (10.9)	22 (91.7)/2 (8.3)	0.721

GEJ, gastroesophageal junction; LVI, lymphovascular invasion; PNI, perineural invasion.

### Survival analyses

**Figure 1:**
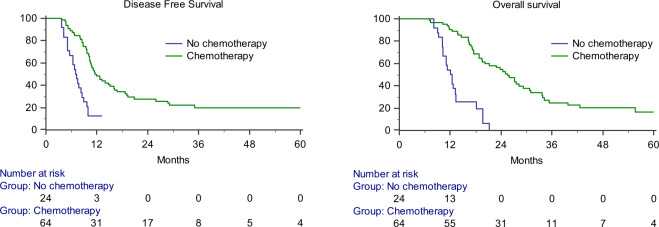
Kaplan-Meier curves of disease-free survival (DFS) and overall survival (OS). Postoperative chemotherapy improved DFS and OS compared to surgery alone in gastric cancer patients with positive cytology but without visible gross peritoneal metastasis.

Postoperative chemotherapy improved DFS and OS compared to surgery alone in gastric cancer patients with positive cytology but without visible gross peritoneal metastasis [chemotherapy (+) vs. surgery alone, median DFS 11.63 months (95% CI 9.28–13.98), vs. 6.98 months (95% CI 5.54–8.42), *p* < 0.001; median OS 25.50 months (95% CI 21.22–29.78) vs. 12.11 months (95% CI 10.47–13.75), *p* < 0.001]. The 1-year DFS rate was 46.9% in the chemotherapy group and 12.5% in the surgery alone group. The 1-year OS rate was 88.7% in the chemotherapy group and 50% in the surgery alone group (Fig. 1). There was no relationship between the survival and the regimen of postoperative chemotherapy.

In univariate analyses of risk factors for DFS, no chemotherapy and N3 status were significantly associated with poor DFS [chemotherapy (−), HR 3.41 (95% CI 1.95–5.95), *p* < 0.001; N3, HR 2.92 (95% CI 1.39–6.10), *p* = 0.004]. In univariate analyses of risk factors for OS, age ≥ 62 years, no chemotherapy, and N3 status were significantly associated with poor OS [age ≥ 62 years, HR 1.66 (95% CI 1.01–2.72), *p* = 0.045; no chemotherapy, HR 5.78 (95% CI 3.12–10.68), *p* < 0.001; N3, HR 2.58 (95% CI 1.23–5.41), *p* = 0.012] (Table 2). In multivariate analyses, no chemotherapy was the strongest independent clinical factor for poorer DFS [HR 3.76 (95% CI 1.95–7.24), *p* < 0.001] and OS [HR 4.37 (95% CI 2.24–8.49), *p* < 0.001].

**Table 2:** Univariate and multivariate analyses of risk factors for disease-free survival and overall survival (n = 88).

Variables (RFS)	Univariate analysis		Multivariate analysis	
	HR (95% CI)	P-value	HR (95% CI)	P-value
Age ≥ 62 years	1.51 (0.94–2.42)	0.088	1.52 (0.93–2.50)	0.097
Male	0.61 (0.37–1.03)	0.063	0.55 (0.31–0.97)	0.04
Tumor location				
GEJ, whole stomach	1.01 (0.63–1.64)	0.957		
Lauren classification				
Non-intestinal (diffuse or mixed)	1.27 (0.77–2.10)	0.348		
Chemotherapy (−)	3.40 (1.95–5.95)	<0.001	3.76 (1.95–7.24)	<0.001
T4	1.81 (0.78–4.20)	0.165	0.91 (0.35–2.33)	0.835
N3	2.92 (1.39–6.10)	0.004	3.65 (1.60–8.35)	0.002
LVI+	0.50 (0.30–0.84)	0.009	0.51 (0.28–0.93)	0.029
PNI+	0.90 (0.43–1.89)	0.786		
**Variables (OS)**	**Univariate analysis**		**Multivariate analysis**	
	**HR (95% CI)**	**P-value**	**HR (95% CI)**	**P-value**
Age ≥ 62 years	1.66 (1.01–2.72)	0.045	1.75 (1.00–3.03)	0.048
Male	0.61 (0.35–1.07)	0.083	0.700 (0.37–1.31)	0.264
Tumor location				
GEJ, whole stomach	0.97 (0.59–1.60)	0.916		
Lauren classification				
Non-intestinal (diffuse or mixed)	1.14 (0.69–1.91)	0.605		
Chemotherapy (−)	5.78 (3.12–10.68)	<0.001	4.37 (2.24–8.49)	<0.001
T4	1.72 (0.74–4.01)	0.21	0.93 (0.31–2.81)	0.9
N3	2.58 (1.23–5.41)	0.012	2.68 (1.03–6.96)	0.044
LVI+	0.47 (0.28–0.79)	0.004	0.37 (0.20–0.71)	0.002
PNI+	0.86 (0.40–1.81)	0.69		

DFS, disease-free survival; OS, Overall survival; GEJ, gastroesophageal junction; LVI, lymphovascular invasion; PNI, perineural invasion.

We also performed binary logistic regression analyses to identify clinical factors associated with positive peritoneal washing cytology (Table 3). For this analysis, we included D2-resected stage II or III gastric cancer patients with negative peritoneal washing cytology (n = 197) (Supplementary Table 1). Gastroesophageal junction cancer and pT4 and pN3 status were significant independent clinical predictors for positive peritoneal washing cytology.

**Table 3:** Univariate and multivariate binary logistic regression analyses to identify clinical factors associated with positive peritoneal washing cytology.

Variables	Univariate analysis		Multivariate analysis	
	OR (95% CI)	P-value	OR (95% CI)	P-value
Age ≥ 62 years	1.02 (0.61–1.68)	0.953		
Male	1.65 (0.94–2.91)	0.08	1.73 (0.94–3.20)	0.079
Tumor location				
GEJ, whole stomach	2.09 (1.21–3.60)	0.008	2.16 (1.19–3.92)	0.012
Lauren classification				
Non-intestinal (diffuse or mixed)	1.37 (0.81–2.30)	0.239	0.93 (0.51–1.67)	0.8
T4	5.06 (2.39–10.68)	<0.001	3.36 (1.47–7.70)	0.004
N3	3.94 (2.14–7.26)	<0.001	3.21 (1.63–6.35)	0.001
LVI+	1.73 (0.98–3.03)	0.057	1.07 (0.57–2.00)	0.847
PNI+	1.96 (0.90–4.28)	0.09	0.98 (0.40–2.83)	0.965

GEJ, gastroesophageal junction; LVI, lymphovascular invasion; PNI, perineural invasion.

The English in this document has been checked by at least two professional editors, both native speakers of English. For a certificate, please see:

http://www.textcheck.com/certificate/YW7ZNg.

## Discussion

The peritoneum is the most common metastatic site of recurrent or initially metastatic gastric cancer. Gastric cancer cells shed into the peritoneal space are believed to develop into peritoneal metastases. The presence of peritoneal metastasis at surgery is a poor prognostic marker, and radical gastrectomy should be reserved only for selected patients with an obstruction or bleeding^17,18^.

Gastric cancer patients with positive cytology but without visible gross peritoneal metastasis are classified as having M1 disease. However, the optimal therapeutic treatment modalities have not been established for these patients. Recent studies have demonstrated that neoadjuvant chemotherapy may improve survival if the cytology results become negative after treatment^19,20^. Another recent study also reported that gastric cancer patients with positive cytology and/or localized peritoneum metastasis who received surgical resection that leaves no macroscopically visible disease benefited from postoperative chemotherapy. They demonstrated median OS was from 24.7 months to 29.5 in the chemotherapy group, and 9.9 months in the no chemotherapy group^21^. In this study, we demonstrated that postoperative chemotherapy also improved OS and DFS compared to surgery alone in this gastric cancer population [chemotherapy (+) vs. surgery alone, median DFS 11.63 months vs. 6.98, *p* < 0.001; median OS 25.50 months vs. 12.11, *p* < 0.001].

Gastrectomy followed by chemotherapy did not result in any survival benefit compared with chemotherapy alone in gastric cancer patients with a visible peritoneal metastasis. Gastrectomy cannot be justified for treating patients with these tumors^18^. However, in this study, all patients underwent radical gastrectomy with D2 lymph node dissection because there were no visible peritoneal metastases at surgery, including other non-curable factors such as distant lymph node metastasis or liver metastasis.

Despite the recently reported benefits of a combination of chemotherapy plus trastuzumab, the prognosis of unresectable advanced or metastatic gastric cancer remains poor. In the ToGA trial, the median OS was 13.8 months in patients assigned to chemotherapy plus trastuzumab, and the median OS was 16.0 months in HER2-overexpressed patients assigned to chemotherapy plus trastuzumab^22^. More recently, a phase II study of nivolumab plus chemotherapy demonstrated promising progression-free survival of about 10 months, and OS was not reached^23^. In future clinical trials or retrospective analyses of chemotherapy, not only conventional chemotherapy, but also chemotherapy plus targeted agent such as trastuzumab or immune-oncologic drug such as nivolumab could be considered in this patient group.

This study has some limitations. This was a retrospective analysis involving a single institution. Second, the gross findings of peritoneal metastasis depended only on the surgeon’s skills and perspective. To some extent this could be subjective. We did not report the adverse events of the chemotherapy regimens; however, all regimens are widely used in a clinical setting, and all toxicities were manageable and did not differ from those reported previously. The gastric cancer treatment modalities used in Eastern and Western countries could be different. Perioperative treatment modalities are used in advanced gastric cancer cases in Western countries^12,24^.

In conclusion, postoperative chemotherapy improves the survival outcome compared to surgery alone in gastric cancer patients with positive peritoneal washing cytology but without visible gross peritoneal metastasis who underwent radical D2 gastrectomy.

## Supplementary information

Supplementary Information.
