# Supplementary material for: Observational Study of Peritoneal Washing Cytology-Positive Gastric Cancer without Gross Peritoneal Metastasis in Patients who Underwent Radical D2 Gastrectomy
Source: Sci Rep. 2020 Jun 12;10:9549. doi: 10.1038/s41598-020-66637-y (PMC7293245; doi:10.1038/s41598-020-66637-y)
Supplement: Supplementary file 1 — Supplementary Information. [file 41598_2020_66637_MOESM1_ESM.docx]

**Original Article**

**Observational Study of Peritoneal Washing Cytology-Positive Gastric Cancer without Gross Peritoneal Metastasis in Patients who Underwent Radical D2 Gastrectomy**

Hyun-Jeong Shim, MD, PhD^1,4*^; Hyeon-Jong Kim, MD^1,4*^; Seung Hyuk Lee MD^1,4^;

Woo-Kyun Bae, MD, PhD^1,4^; Eu-Chang Hwang, MD^2,4^; Sang-Hee Cho, MD, PhD^1,4^;

Ik-Joo Chung, MD, PhD^1,4^; Hyun-Jin Bang MD^1,4^; Jun Eul Hwang MD, PhD^1,4^

*Department of Hematology-Oncology^1^, Department of Urology^2^,*

*Chonnam National University Medical School and Hwasun Hospital, Gwangju, Korea^4^*

*Hyun-Jeong Shim and Hyeon-Jong Kim contributed equally to this work as first co-authors

***Corresponding author and request for reprint***

Jun Eul Hwang, MD, PhD, Department of Hematology-Oncology,

Chonnam National University Hwasun Hospital, 322, Seoyang-ro, Hwasun-eup, Hwasun-gun, Jeonnam, 58128, South Korea. Tel: +82-61-379-7622; Fax: +82-61-379-8019; E-mail: hjunyl@naver.com

Supplementary Table 1. D2-resected stage II / III gastric cancer patients with negative peritoneal washing cytology

| Variables, n (%) | Cytology (-) |
| --- | --- |
|  | n = 197 (%) |
| Ages |  |
| <61 | 97 (49.2( |
| ≥61 | 100 (50.8) |
| Sex |  |
| Male | 127 (64.5) |
| Female | 70 (35.5) |
| Tumor location |  |
| GEJ, whole stomach | 44 (22.3) |
| body, antrum | 153 (77.3) |
| Tumor grade |  |
| well/moderate differentiated | 53 (26.9) |
| poorly/un-differentiated | 144 (73.1) |
| Lauren classification |  |
| intestinal | 84 (42.6) |
| non-intestinal (diffuse or mixed) | 133 (57.4) |
| T stage |  |
| T1/2/3 | 72 (36.5) |
| T4 | 125 (63.5) |
| N stage |  |
| N0/1/2 | 92 (46.7) |
| N3 | 105 (53.3) |
| LVI+ / LVI- | 125 (63.5) / 72 (36.5) |
| PNI+ / PNI- | 161 (81.7) / 36 (18.3) |

*GEJ*, gastroesophageal junction; *LVI*, lymphovascular invasion; *PNI*, perineural invasion.
